# Supplementary material for: DoxoDB: A Database for the Expression Analysis of Doxorubicin-Induced lncRNA Genes
Source: Noncoding RNA. 2023 Jul 13;9(4):39. doi: 10.3390/ncrna9040039 (PMC10366827; doi:10.3390/ncrna9040039)
Supplement: Supplementary file 1 [file ncrna-09-00039-s001.zip › Supplementary_Figures.pdf]

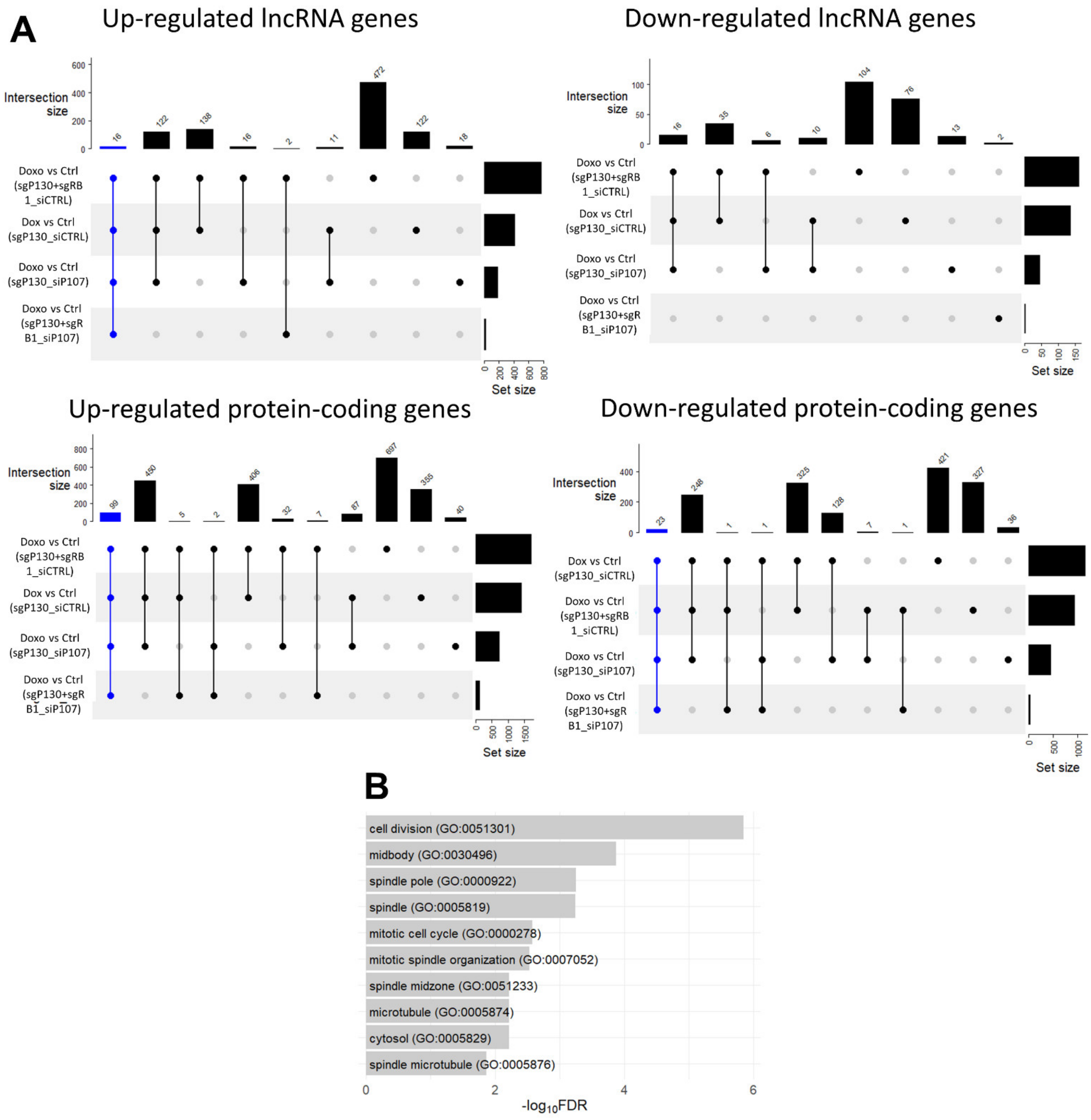

Figure S1. Shared DEGs between DOX-treated sgP130_siCTRL, sgP130_siP107, sgP130+sgRB1_siCTRL, and sgP130+sgRB1_siP107 HFF cells compared to the control cells. (A) UpSet plots of up- and down-regulated lncRNA and protein-coding genes between DOX-treated cells compared to control cells. (B) Enriched GO terms for the shared down-regulated protein-coding genes.

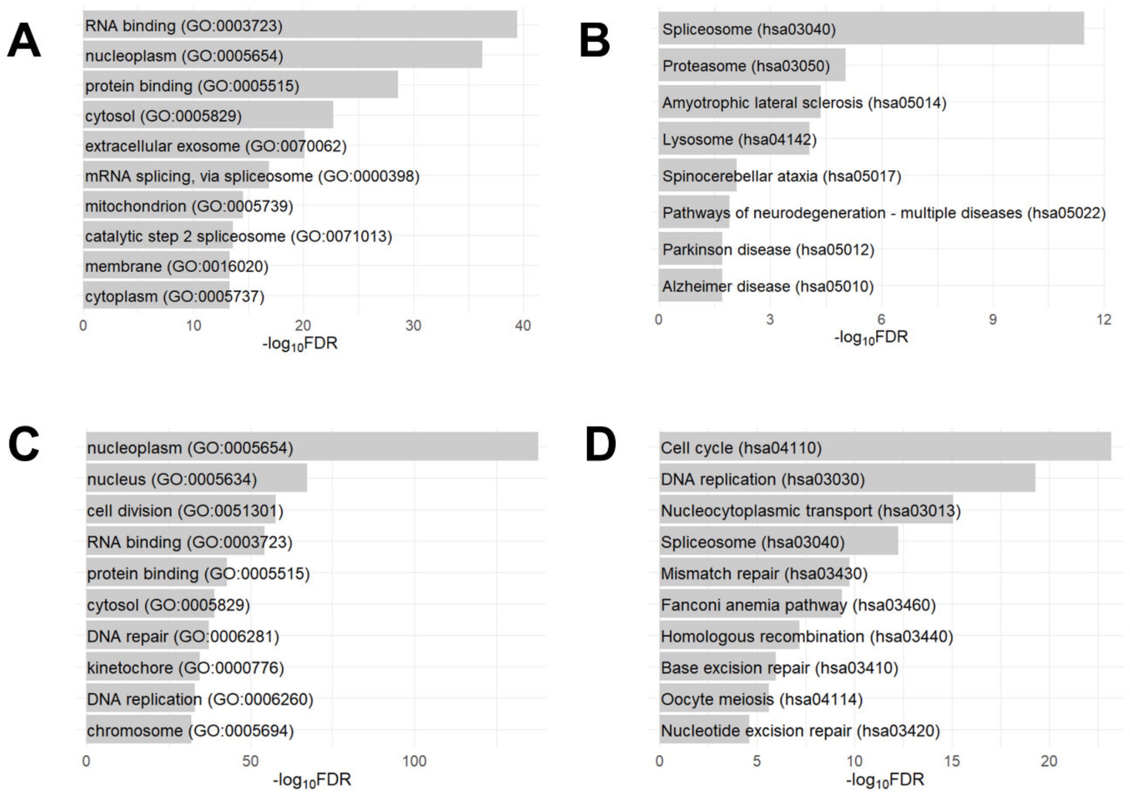

Figure S2. Expression correlation analysis of the differentially expressed lncRNA genes and protein-coding genes in DOX-induced senescent *PD-L1+* and *PD-L1-* cells compared to the control cells. (A) Enriched GO terms (top 10) and (B) KEGG pathways for the most significantly correlated protein-coding genes to the shared up-regulated lncRNA genes. (C) Top 10 enriched GO terms and (D) KEGG pathways for the most significantly correlated protein-coding genes to the shared down-regulated lncRNA genes.

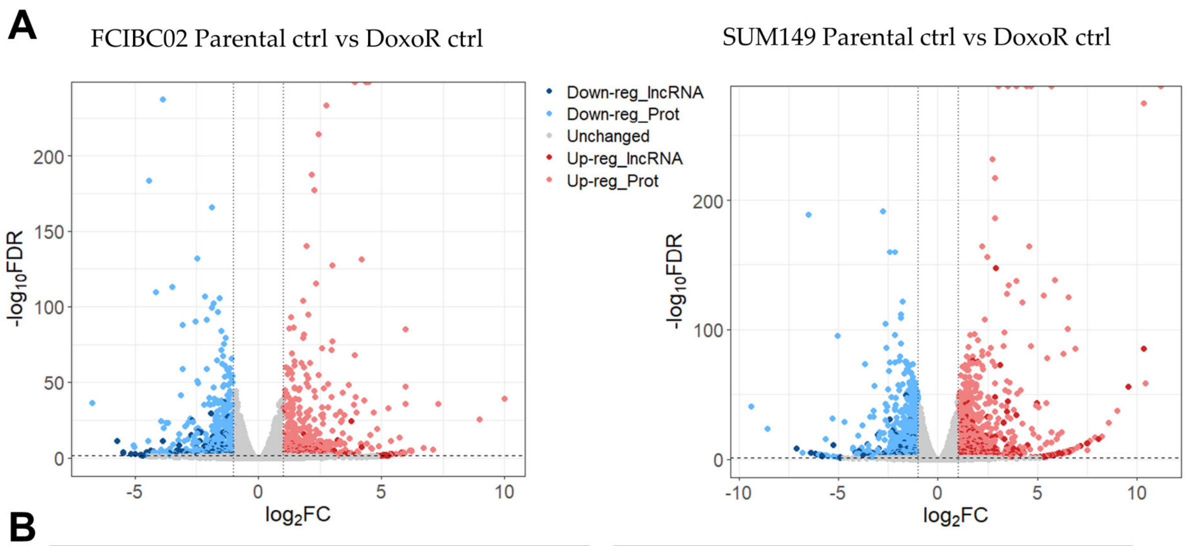

B

Up-regulated	FCIBC02 Parental ctrl vs DoxoR ctrl	SUM149 Parental ctrl vs DoxoR ctrl
Protein-coding	642	861
lncRNA	200	421
Others	32	70
Total	874	1352

Down-regulated	FCIBC02 Parental ctrl vs DoxoR ctrl	SUM149 Parental ctrl vs DoxoR ctrl
Protein-coding	434	722
lncRNA	137	162
Others	206	87
Total	777	971

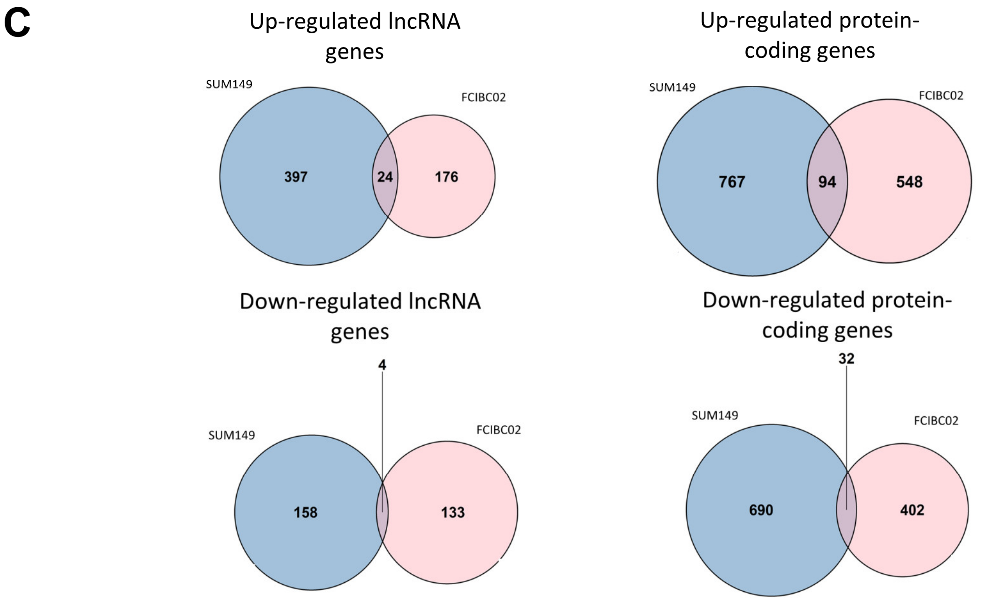

Figure S3. RNA-seq data analysis of DOX-resistant IBC cells (FCIBC02 and SUM149 cells) compared to control cells. (A) Volcano plot of differentially expressed genes based on the threshold values of 2-fold change and FDR < 0.05. (B) Number of differentially expressed lncRNA, protein-coding and other types of genes. (C) Venn diagrams of shared up- and down-regulated lncRNA and protein-coding genes between DOX-resistant IBC FCIBC02 and SUM149 cells compared to the control cells (*p*-value < 0.0001).
